# Energy Harvesting System Whose Potential Is Mapped with the Modified Fibonacci Function

**DOI:** 10.3390/s23146593

**Published:** 2023-07-21

**Authors:** Jerzy Margielewicz, Damian Gąska, Grzegorz Litak, Jacek Caban, Agnieszka Dudziak, Xiaoqing Ma, Shengxi Zhou

**Affiliations:** 1 Faculty of Transport and Aviation Engineering, Silesian University of Technology, Krasińskiego 8, 40-019 Katowice, Poland; jerzy.margielewicz@polsl.pl (J.M.); damian.gaska@polsl.pl (D.G.); 2 Faculty of Mechanical Engineering, Lublin University of Technology, Nadbystrzycka 36, 20-618 Lublin, Poland; g.litak@pollub.pl (G.L.); j.caban@pollub.pl (J.C.); 3 Faculty of Production Engineering, University of Life Sciences in Lublin, Głęboka 28, 20-612 Lublin, Poland; 4 School of Aeronautics, Northwestern Polytechnical University, Xi’an 710072, China; mxq19911101@mail.nwpu.edu.cn (X.M.); zhoushengxi@nwpu.edu.cn (S.Z.)

**Keywords:** transient chaos, bifurcations, energy effectiveness, multistable energy harvester

## Abstract

In this paper, we compare three energy harvesting systems in which we introduce additional bumpers whose mathematical model is mapped with a non-linear characteristic based on the hyperbolic sine Fibonacci function. For the analysis, we construct non-linear two-well, three-well and four-well systems with a cantilever beam and permanent magnets. In order to compare the effectiveness of the systems, we assume comparable distances between local minima of external wells and the maximum heights of potential barriers. Based on the derived dimensionless models of the systems, we perform simulations of non-linear dynamics in a wide spectrum of frequencies to search for chaotic and periodic motion zones of the systems. We present the issue of the occurrence of transient chaos in the analyzed systems. In the second part of this work, we determine and compare the effectiveness of the tested structures depending on the characteristics of the bumpers and an external excitation whose dynamics are described by the harmonic function, and find the best solutions from the point view of energy harvesting. The most effective impact of the use of bumpers can be observed when dealing with systems described by potential with deep external wells. In addition, the use of the Fibonacci hyperbolic sine is a simple and effective numerical tool for mapping non-linear properties of such motion limiters in energy harvesting systems.

## 1. Introduction

Vibration energy harvesting technology is a popular way to power small autonomic sensors and system monitoring in the emerging technologies [1,2,3,4,5,6]. On account of their high energy density, piezoelectric transducers are proposed to convert mechanical vibration energy into electrical power. In many proposed energy harvesting systems, ambient conditions are used as a source of vibration and the device is composed of a mechanical resonator and a piezoelectric part where its deformations produce electromotive force [6,7,8,9]. Unfortunately, any simple linear system can only work well at its resonance frequency. To overcome this limitation, nonlinear devices are proposed by many scholars [10]. The main directions of the modeling and laboratory studies include multi-stable [11,12] and/or impacting devices [13,14,15,16]. Additional possibilities are using multiple-degree-of-freedom systems [17,18,19,20,21], self-adapting the resonator to the excitation conditions [22].

We have been observing the dynamic development of research on this issue since the appearance of the first papers on non-linear systems [23,24]. The advantage of such systems enables effective energy generation in a wide range of excitation parameters (frequencies and amplitudes), as opposed to linear systems tuned only to the resonant frequency of the excitation source. A typical design form of kinetic energy harvesters is made of a flexible cantilever beam with piezoelectric materials glued on [25,26,27]. Mechanical vibrations of the beam, excited by the source of excitation, cause its deformation, and piezoelectric materials convert kinetic energy into electric energy. Non-linearities are introduced by the system of magnets [28,29] placed on the rigid frame and the end of the cantilever beam, which can construct bistable (BEH), tristable (TEH) and multistable systems [30]. Other ways to introduce non-linearity to the system are introducing spring elements [31] or a buckling-possible design [32]. The amount of energy obtained is small, but it is sufficient to power various sensors monitoring the operation of machines [33].

Issues related to the use of bumpers (motion limiters, amplitude limiters) in kinetic energy harvesting systems have already been the subject of scientific publications [34,35,36]. The main goal was to find a method for broadening the operating frequency range. The wideband frequency responses of an EH system with limiters on one side and two sides was the subject of Liu et al.’s paper [37]. The laboratory experiment show that the operating frequency bandwidth was broadened together with the corresponding optimal power ranges. Similar studies were carried out in [38], which proposed a two-degree-of-freedom piecewise linear piezoelectric energy harvester by combining the multimodal harvesting technique and nonlinear method. Hu et al. [39] proposed a parametric study of stopper distance for the piecewise linear stiffness model on the energy harvesting performance in terms of both the bandwidth and open-circuit voltage output. Laboratory experiments and FEM model simulations on the use of bumpers in energy harvesting systems were also carried out in the papers [40,41,42].

In this paper, we propose a system with multistable potential and additional impacts. The impact interaction is modeled with the Fibonacci hyperbolic sine. The simulation results are provided to compare the system responses for several cases with and without impacts. Finally, the effectiveness of the corresponding system is studied.

## 2. Mathematical Model Formulation

**Figure 1:**
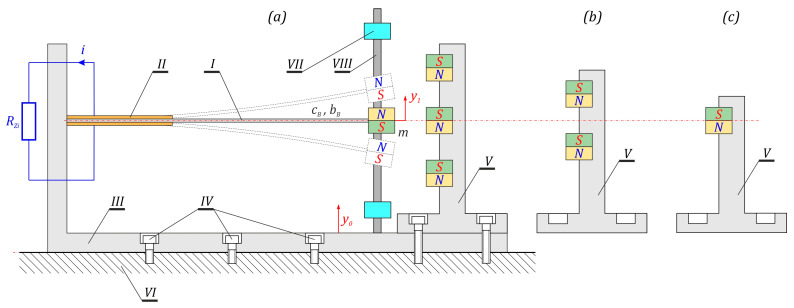
Schematic diagram and phenomenological model of the tested energy harvesting systems: (**a**) structural form of the system with 4 potential wells, (**b**) magnet system for 3 potential wells, (**c**) magnet system for 2 potential wells.

The subject of model tests is the design solution of the energy harvesting system, the dynamics of which are based on the potential induced by permanent magnets fixed in a rigid frame V. The dynamic properties of the system are tested for three symmetrical potentials with four (Figure 1a), three (Figure 1b) and two wells (Figure 1c). In the conducted numerical experiments, the mechanical limiters (bumpers) VII which can limit the movement of the flexible cantilever beam I are taken into account. During the computer simulations, the possibility of adjusting the “zone of free motion” of the flexible cantilever beam is assumed. At this point, we explain that the term “zone of free motion” means beam vibrations in the plane located between the upper and lower limiters VII. The beam element I on which the piezoelectric transducers II are glued is fixed in a rigid, non-deformable frame III, which is attached to the mechanically vibrating object VI by means of screws IV. The general description of the energy harvesting system is only intended to sketch and illustrate the essence of the research object. It does not refer to a specific design solution that must meet many technical requirements.

**Figure 2:**
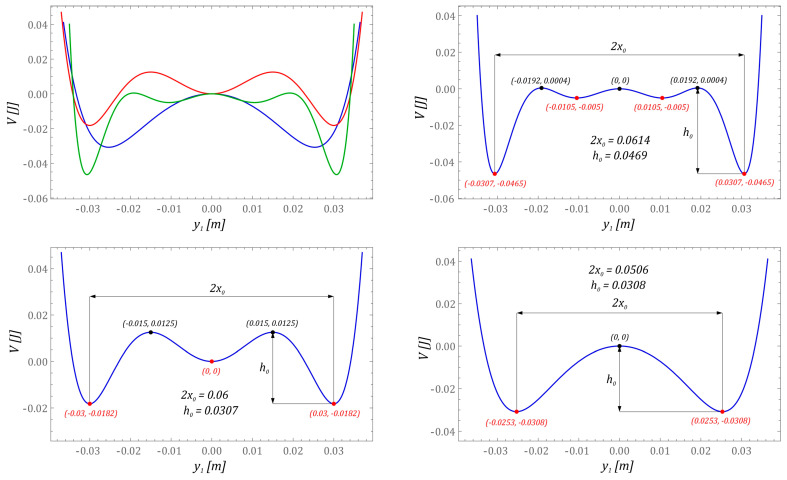
Properties of the investigated functions of energy potentials for the same distance between local minima of external potential wells and depth.

The inclusion of bumpers during model tests is intended to limit the displacement of the cantilever beam. In addition, their presence in the design of the energy harvesting system is also directly related to increasing the durability of the vibrating element on which the piezoelectric transducers are glued. External mechanical vibrations of high amplitude affecting the system over time can lead to device failure, which is mainly caused by the phenomenon of material fatigue [43,44]. Examples of potential characteristics reflecting the cause-and-effect relationships of permanent magnet configurations of the tested design solutions of energy harvesting systems are shown in the diagrams (Figure 2).

During the identification of potentials, comparable distances between local minima of external wells are assumed, with the estimated average value being at the level of 2*x*_0_ ≈ 0.057 ± 0.0058 m. It should be noted that the maximum heights of potential barriers should have similar values of *h*_0_ ≈ 0.036 ± 0.009 m. The potentials identified in this way are transformed into a dimensionless form. Based on these, the quantitative and qualitative numerical simulations are carried out in the field of energy harvesting effectiveness.

### 2.1. Model of the Flexible Vibration Limiter

**Figure 3:**
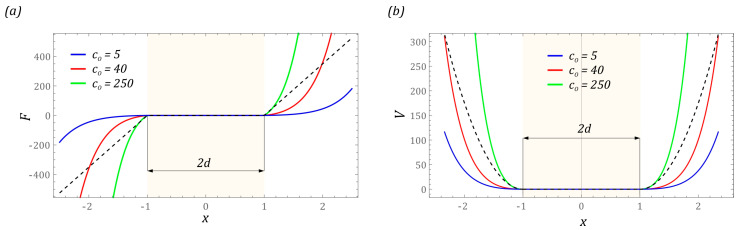
Influence of stiffness *c*_0_ on the shape of: (**a**) mechanical characteristics; (**b**) potential. The sand color marks the area where we are dealing with unlimited vibrations of the flexible cantilever beam. Black dashed lines represent linear model of the limiter.

In the identified configurations of the tested design solution of the energy harvesting system, solutions characterized by an asymmetric arrangement of the potential wells dominate (Figure 3). Moreover, in the analyzed set of configurations, there are very often potentials whose external wells are characterized by large or very large depth. If the structure is characterized by such mapped potential, then there is a very high probability that the operating point of the system will be in the area of its attraction. As a result, the effectiveness of energy harvesting is significantly reduced. Minimizing the impact of deep potential wells can be limited by using mechanical limiters. The use of such bumpers has its disadvantages, which boil down to reducing the potential width and thus limiting the amplitudes of large orbits circulating the potential well. Nevertheless, the use of flexible motion bumpers can significantly improve energy harvesting effectiveness. Numerous mathematical models are used to numerically represent the mechanical properties of flexible limiters. The most popular of them is a model based on a non-smooth piecewise-defined function, also called a function with a dead zone [45,46]:

(1) $$f\left(q\right)=\begin{array}{ccc} \left\{\begin{array}{cc} q+d, & q<-d,\\ 0, & -d\leq q\leq d,\\ q-d, & q>d. \end{array}\right. & \overset{\begin{array}{cc} x=\frac{q}{x_{0}}, & \eta =\frac{d}{x_{0}} \end{array}}{\Rightarrow } & x_{0}f\left(x\right)=\left\{\begin{array}{cc} x+\eta , & x<-\eta ,\\ 0, & -\eta \leq x\leq \eta ,\\ x-\eta , & x>\eta . \end{array}\right. \end{array}$$

The popularity of such a description is mainly determined by the simplicity of the mathematical notation. Much better results can be achieved when carrying out numerical simulations by mapping the “zones of free motion” with a continuous model. The reason is that the piecewise function (1) extends the time of computer simulations, which is caused by the need to verify the operating point with logical conditions in each calculation step.

(2) $$\begin{array}{ccc} f\left(q\right)=\left(q+\frac{\left|q-d\right|-\left|q+d\right|}{2}\right) & \overset{\begin{array}{cc} x=\frac{q}{x_{0}}, & \eta =\frac{d}{x_{0}} \end{array}}{\Rightarrow } & x_{0}f\left(x\right)=\left(x+\frac{\left|x-\eta \right|-\left|x+\eta \right|}{2}\right) \end{array}.$$

From an engineering point of view, limiters have different mechanical properties. Depending on the construction and the materials used for the flexible washer, there are buffers with linear and non-linear characteristics. To account for the simplification of the notation of equations while maintaining the essence of the phenomena occurring in systems with motion limiters, we map the mathematical model of the bumpers with a non-linear characteristic based on the hyperbolic sine Fibonacci function [47,48]. By doing such, it is possible to easily reproduce the soft and hard mechanical characteristics of washers and the stiffening effect of the material showed by a rapid increase in stress with a relatively small change in its deformation. The mathematical model of the limiters that we used during the computer simulations takes the following form:

(3) $$F_{O}=c_{O}\cdot sFh{\left(q\right)}^{2}\cdot f(q).$$

where

$Fh(q)=\frac{2}{\sqrt{5}}sinh\left(2ln\left(\varphi \right)q\right)$, *φ* ≈ 1.61803—golden ratio;
$c_{O}$—parameter defining the variable stiffness of the bumpers.

In general, the formulated mathematical model of the bumper is the product of the classical model with the dead zone and the square of the Fibonacci hyperbolic sine. At this point, it should be noted that, from a technical point of view, the stiffness of the bumpers (limiters) can be shaped in any way. First of all, it is determined by the applied construction solution. If the motion limiters are to be characterized by linear characteristics and low stiffness values, then their construction solutions can be based on flexible cantilever beams or beams fixed on both sides. From the technical point of view, the easiest way to characterize bumpers with high stiffness is in the form of rods. Such limiters equipped with flexible tips made of plastics or silicones are described by non-linear characteristics which can be represented by the proposed function (3). The impact of the non-linear limiter on the mechanical characteristics and potential shape are shown in the diagrams (Figure 3). The presented functions are plotted against the background of the linear model of the bumper whose characteristics are plotted with black dashed lines. The sand color marks the area where we are dealing with unlimited vibrations of the flexible cantilever beam of the tested design solution of the energy harvesting system.

**Figure 4:**
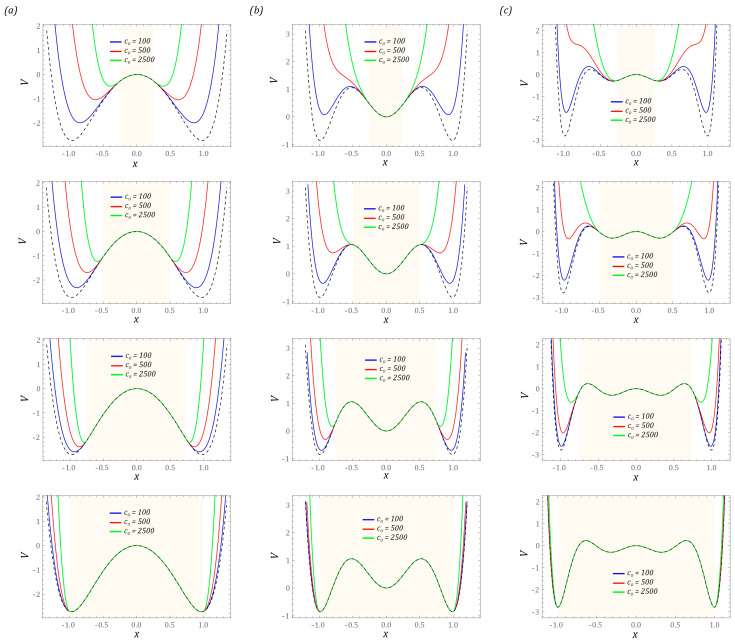
The influence of the width of the “zone of free motion” and stiffness on the following potential characteristics: (**a**) two-well, (**b**) three-well, (**c**) four-well. The sand shadow marks the area where we are dealing with unlimited vibrations of the flexible cantilever beam. Black dashed lines represent the linear model of the limiter.

The graphs show that increasing the hardness of the flexible tips reduces the distance measured between the external potential barriers. At this point, it is worth mentioning that the hardness of flexible tips is directly correlated with their stiffness. In other words, the harder the material is, the greater its stiffness is. The images of the exemplary characteristics suggest that, from the point view of energy harvesting, it is reasonable to use soft tips (washers). The orbits of stable periodic solutions reach larger vibration amplitudes, as a consequence of which the effectiveness of energy harvesting is improved. The impulse to undertake model tests is the assessment of the mechanical properties of the bumpers and the width of the “zone of free motion” on the effectiveness of energy generation. Numerical simulations are carried out for the four-well potential, whose external wells are much deeper than the internal wells. Example diagrams showing the influence of the linear and non-linear motion limiter on the mechanical characteristics and potential are shown in the diagrams (Figure 4).

The presented results of the model tests indicate a significant impact of the bumper stiffness on the potential width. At the same time, its value decreases with increasing stiffness of the bumper (Figure 4a). This situation takes place regardless of the tested design solutions of energy harvesting systems. Analogous conclusions can be drawn with regard to the location of motion limiters. For example, for narrow “zones of free motion”, the use of rigid bumpers limits the number of wells. On the other hand, bumpers characterized by high susceptibility do not significantly reduce the height of the potential barrier. This situation is particularly visible in relation to multi-well systems (Figure 4b,c).

### 2.2. Dimensionless Mathematical Model of Energy Harvesting Systems

Differential equations of the motion of non-linear dynamical systems can be derived by various methods: classical and non-classical [49,50,51]. Regardless of the method used, the mathematical model is ultimately represented by the same structure of differential equations. In the numerical experiments, it is assumed that the system is affected by an external excitation whose dynamics are described by the harmonic function $y_{0}=Asin\left(\omega_{W}t\right)$. The general structure of the system of differential equations representing the dynamics of the analyzed design solutions of energy harvesting systems takes the following form:

(4) $$\left\{\begin{array}{l} m\frac{d^{2}y_{1}}{dt^{2}}+b_{B}\left(\frac{dy_{1}}{dt}-\frac{dy_{0}}{dt}\right)+c_{B}\left(y_{1}-y_{0}\right)-c_{1}\left(y_{1}-y_{0}\right)+c_{2}{\left(y_{1}-y_{0}\right)}^{3}\\ -c_{3}{\left(y_{1}-y_{0}\right)}^{5}+c_{4}{\left(y_{1}-y_{0}\right)}^{7}+c_{O}f\left(y_{1}-y_{0}\right)+k_{P}v=0,\\ C_{P}\frac{dv}{dt}+\frac{1}{R_{P}}v-k_{P}\left(\frac{dy_{1}}{dt}-\frac{dy_{0}}{dt}\right)=0. \end{array}\right.$$

Taking into account the efficient performance of computer simulations, the mathematical model (4) can be transformed into a dimensionless form. For this purpose, a new coordinate representing the difference of displacements *q* = *y*_1_ − *y*_0_ is introduced. Mathematical models provided by systems of non-linear differential equations reflect the dynamics of the two-well (5), three-well (6) and four-well (7) systems, respectively.

(5) $$\left\{\begin{array}{l} \ddot{x}+\delta \dot{x}+x\left(1+\mu_{1}\left(\beta x^{4}-1\right)\right)+\mu_{2}f\left(x\right)+\theta u=\omega^{2}psin\left(\omega \tau \right),\\ \dot{u}+\kappa u-\dot{x}=0, \end{array}\right.$$

(6) $$\left\{\begin{array}{l} \ddot{x}+\delta \dot{x}+x\left(1+\mu_{1}\left(\beta x^{4}-\alpha x^{2}+1\right)\right)+\mu_{2}f\left(x\right)+\theta u=\omega^{2}psin\left(\omega \tau \right),\\ \dot{u}+\kappa u-\dot{x}=0, \end{array}\right.$$

(7) $$\left\{\begin{array}{l} \ddot{x}+\delta \dot{x}+x\left(1+\mu_{1}\left(\gamma x^{6}-\beta x^{4}+\alpha x^{2}-1\right)\right)+\mu_{2}f\left(x\right)+\theta u=\omega^{2}psin\left(\omega \tau \right),\\ \dot{u}+\kappa u-\dot{x}=0, \end{array}\right.$$

where

$$\begin{array}{c} \begin{array}{cccccccc} \omega_{0}^{2}=\frac{c_{B}}{m}, & \tau =\omega_{0}t, & x=\frac{q}{x_{0}}, & \delta =\frac{b_{B}}{\omega_{0}m}, & \mu_{1}=\frac{c_{1}}{c_{B}}, & \mu_{2}=\frac{c_{O}}{c_{B}}, & \eta =\frac{d}{x_{0}}, & \alpha =\frac{c_{2}x_{0}^{2}}{c_{1}}, \end{array}\\ \begin{array}{ccccccc} \beta =\frac{c_{3}x_{0}^{4}}{c_{1}}, & \gamma =\frac{c_{4}x_{0}^{6}}{c_{1}}, & \omega =\frac{\omega_{W}}{\omega_{0}}, & p=\frac{A}{x_{0}}, & u=\frac{C_{P}}{k_{P}x_{0}}v & \kappa =\frac{1}{\omega_{0}C_{P}R_{P}}, & \theta =\frac{k_{P}^{2}}{m\omega_{0}^{2}C_{P}}. \end{array} \end{array}$$

The parameter values which are used in the numerical simulations when we analyze the dynamic properties of the considered systems are summarized in Table 1.

The derived mathematical model is a formal basis for conducting quantitative and qualitative model tests in the field of evaluating the effectiveness of harvesting energy from vibrating mechanical devices.

## 3. Results of Numerical Calculations

### 3.1. Periodicity of Solutions and Transient Chaos

**Figure 5:**
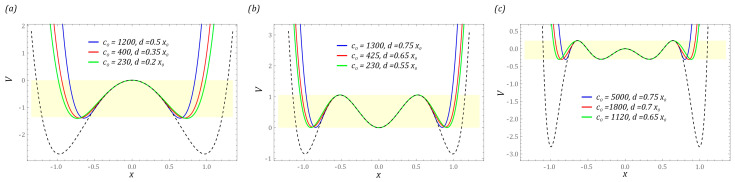
The impact of the stiffness and position of the bumper (motion limiter) on potential characteristics in the system with (**a**) two wells, (**b**) three wells, (**c**) four wells. The assumed maximum heights of the potential barrier are highlighted in sand shadow. The characteristic of the potential without motion limiters is drawn with a dashed black line.

The results presented in the graphs (Figure 4) showing the impact of the bumper characteristics on the potential clearly indicate the possibility of a direct impact on the shape of the potential barrier—in particular, the depth of the well. In the later part of the paper, computer simulations are carried out, the purpose of which is to assess the dynamic properties of the tested design solutions of energy harvesting systems. Considering their performance, the characteristics of the motion limiters are assumed in such a way that the potential depth of the two-well system with bumpers is equal to half of the potential depth without it. In the case of structural solutions whose potentials are provided by three and four wells, the maximum height of the potential barrier of the system with limiters is equal to the depth of the shallowest well. The assumptions adopted in this way regarding the height of the maximum potential barrier directly determine the minimum and maximum width of the “zone of free motion”. For each analyzed construction solution, the assumed maximum heights of the potential barrier are highlighted in yellow. The width of the “zone of free motion” is determined based on the equivalent stiffness of the bumpers. Their values are selected in such a way as to ensure the assumed maximum height of the potential barrier. The characteristics of the potentials analyzed below are marked in blue, red and green against the background of the potential corresponding to the solution without motion limiters, which is drawn with a dashed line in black (Figure 5).

**Figure 6:**
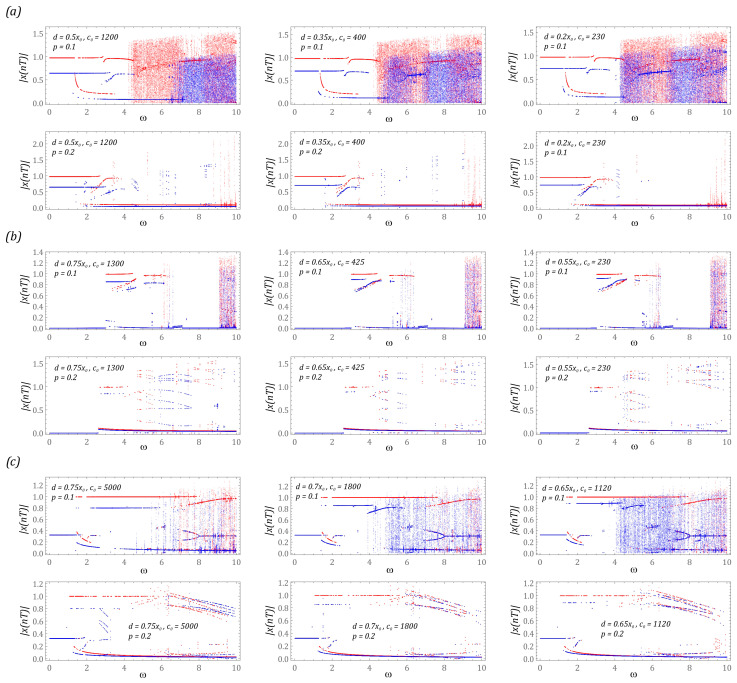
The results of computer simulations showing periodic solutions with and without motion limiters in systems with the following potential: (**a**) two-well, (**b**) three-well, (**c**) four-well. The red color corresponds to the energy harvester with bumpers, and the blue color corresponds to the energy harvester without limiters.

In the first stage of the model tests, the influence of the equivalent stiffness of the bumpers and their position on the structure of steady-state bifurcation diagrams is presented (Figure 6). During their plotting, the dimensionless frequency of external vibrations affecting the tested construction solutions is selected as the control parameter. As a result, we have the opportunity to assess the energy harvesting effectiveness in a wide range of frequency variability, which is carried out with a resolution of 1200 periods of excitation. On the other hand, the dynamic properties of steady states are considered in the time window, the length of which is assumed to be 50 periods of mechanical vibrations affecting the tested structural design.

One of the basic applications of bifurcation diagrams comes down to the identification of zones of occurrence of periodic and chaotic solutions. From a theoretical point of view, bifurcation diagrams can be plotted using various algorithms. The most popular approach is based on the identification of local minima and maxima in the time sequence of the generalized coordinate. In our research, we use an alternative approach, the formal basis of which is the Poincaré section. We made such a decision because the periodicity of the excited solutions determined on the basis of the time sequence does not always provide reliable results. In this context, bifurcation diagrams drawn on the basis of Poincaré sections are much more accurate. At this point, it should be mentioned that any attempt to estimate the size of the orbit of the solution on the basis of the bifurcation diagram drawn in this way will basically fail in advance. This situation occurs because the points in the diagram de facto represent the points of intersection of the phase flow with the control plane, and in principle they never coincide with the points corresponding to the maximum displacements of the orbit.

The identified steady-state bifurcation diagrams indicate the existence of areas of chaotic solutions. In particular, it is clearly visible in the case of the system based on the two-well potential (Figure 6a), when the energy harvesting system is affected by an external load with a small amplitude (*p* = 0.1). In the case of a level of mechanical vibrations twice as high, we are basically dealing only with periodic solutions. A similar situation occurs with regard to systems based on the three- and four-well potential. In the example of the three-well system (Figure 6b), chaotic solutions are excited in the range of high values of the dimensionless excitation frequency ω > 9. If the dynamics of the energy harvesting system are based on the four-well potential (Figure 6c), the zones of chaotic solutions are excited at significantly lower frequencies ω > 5. It is worth noting that only in relation to the three-well potential can it be claimed that the chaotic responses of structures with and without bumpers coincide. In the other two cases, these zones are located in different bands. Detailed numerical experiments are carried out for selecting values of the dimensionless frequency of mechanical vibrations which affect the systems, and the results have shown that the unstable chaotic solutions over time are attracted by stable periodic responses. The presence of the phenomenon of transient chaos is indicated by clear branches of bifurcation diagrams occurring in these bands. It is possible to distinguish them by assigning a specific transparency to the points in the diagrams. When several points overlap, the resultant point is plotted in a darker shade. These branches are very visible in the bifurcation diagrams that have been identified for systems with a two-well potential (Figure 6a) and a four-well potential (Figure 6c).

**Figure 7:**
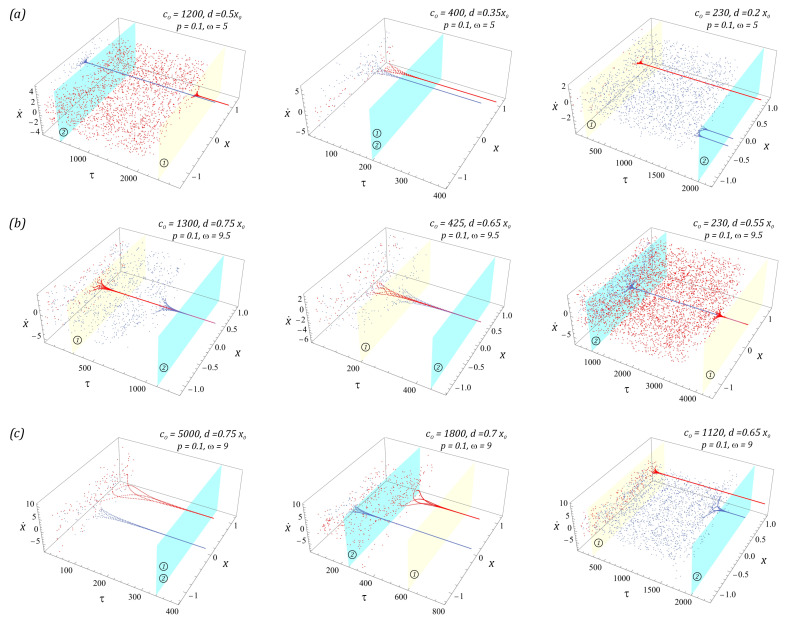
Simulation results showing the phenomenon of unstable chaos in the system: (**a**) two-well, (**b**) three-well, (**c**) four-well, in the form of three-dimensional Poincaré sections versus dimensionless time. The system parameters are included in the figures. The light blue and yellow surfaces define the times when the chaotic response is absorbed into a stable periodic orbit. Notation of individual surfaces assigned to systems: without limiters ① and with them ②.

Examples of cases illustrating the phenomenon of transient chaos identified in the analyzed design solutions of the energy harvesting systems are shown in the diagrams (Figure 7).

To visualize unstable chaotic solutions, we use the procedure based on the Poincaré section. From the mathematical point of view, the graphs (Figure 7) are de facto classic Poincaré sections, which have been depicted in three-dimensional form. Such a representation makes it possible to determine the time after which unstable chaotic solutions are extinguished. The light blue and yellow planes define the times when the chaotic response is absorbed into a stable periodic orbit. At this point, we would like to indicate the individual planes assigned to systems without bumpers ① and with them ②. Such analyses are important for this reason, because they clearly determine what kind of solution we are dealing with. In the graphs (Figure 7), transient chaotic solutions are attracted by periodic orbits with a periodicity of 1T. On the other hand, in the two-well system with soft bumpers and a narrow “zone of free motion” (*c*_0_ = 230, *d* = 0.2*x*_0_), the chaotic solution is attracted by a periodic orbit with a periodicity of 2T. It is worth noting that the use of limiters does not have a significant effect on the excitation of unstable or unpredictable solutions.

**Figure 8:**
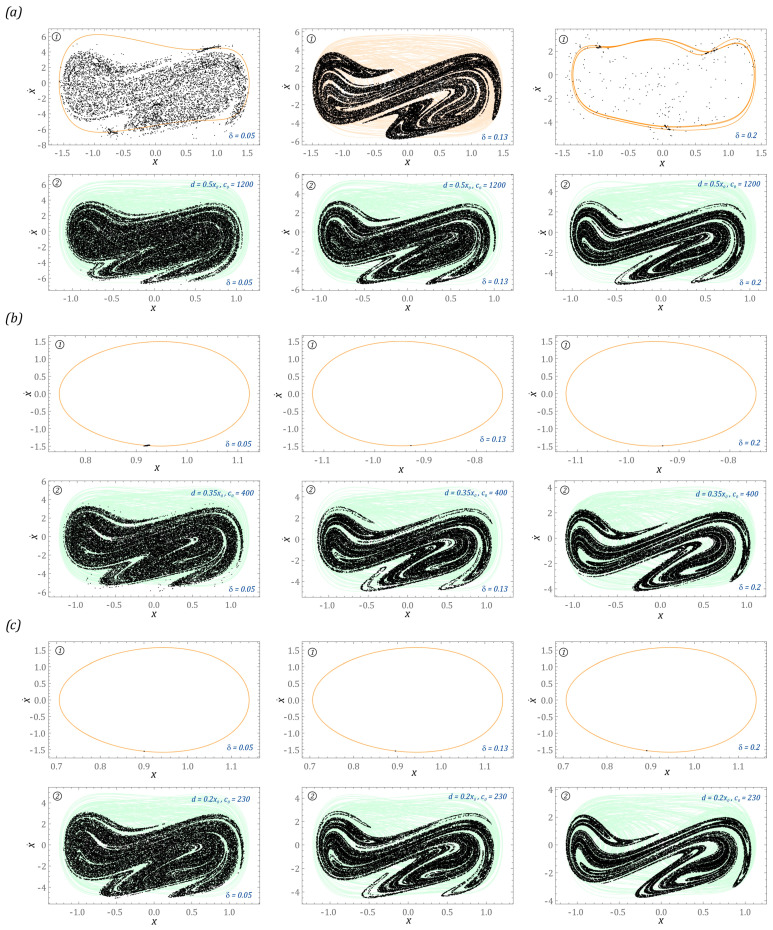
The results of computer simulations showing periodic solutions with and without motion limiters in systems with potential (**a**) *p* = 0.1, ω = 9.25, (**b**) *p* = 0.1, ω = 8, (**c**) *p* = 0.1, ω = 7.25. The shadows over the Poincare points (in black) indicate the ranges of phase portraits. The individual planes have been assigned to systems without limiters ① and with them ②. The system parameters are included in the figures.

Permanent chaotic solutions are very rare and have been identified in the case of a system with a two-well potential. Example images of chaotic solutions showing the impact of damping on them are shown in the graphs (Figure 8).

In this system, the geometry of chaotic attractors is represented by a random distribution of Poincaré cross-section points in a limited area of the phase plane (Figure 8a). The fuzzy structure of the chaotic attractor is mainly determined by the small value of the factor responsible for the dissipation of energy in the system. In the case of a flexible cantilever beam made of a material exhibiting higher dissipation properties, its geometry begins to take on a clear picture. It is also worth mentioning that, by increasing the damping of the energy harvesting system, the zones in which unpredictable solutions are excited are limited. From an engineering point of view, this can be achieved by making a flexible cantilever beam out of a composite material. It is worth bearing this in mind when designing the structure because, in the areas of chaotic solutions, there is a loss of the system’s ability to harvest energy [26,31].

Modification of the damping coefficient in the system is also associated with a change in the nature of the system response. In particular, this applies to solutions excited in the system without limiters. In the example (Figure 8a), we deal with an unstable chaotic solution, which, over time, is attracted to a sTable 1T-periodic orbit. Increasing the dimensionless damping coefficient to the value δ = 0.13 causes a stable chaotic solution to be excited in the system. If the value of the dimensionless damping coefficient increases to the level δ = 0.2, then the response of the system ① in steady state is a periodic orbit characterized by high periodicity. In the example presented in the graphs (Figure 8b), we deal with periodic solutions with a periodicity of 1T. On the other hand, a change in the value of the energy dissipation affects the location of the solution. In other words, the response of the system is located in one of the external potential wells. In the third example (Figure 8c), the change in the damping coefficient does not affect the nature of the excited solutions.

### 3.2. Effectiveness of Energy Harvesting

**Figure 9:**
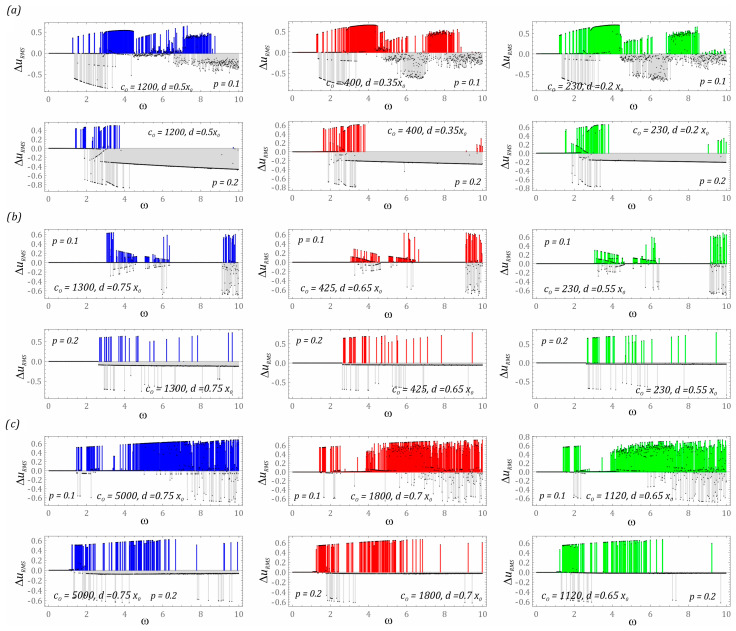
The results of computer simulations showing the impact of the stiffness and position of the bumper on the effectiveness of energy harvesting in the system: (**a**) two-well, (**b**) three-well, (**c**) four-well. The colors of the plotted diagrams directly correlate with the corresponding characteristics of the potentials, which are depicted in the graphs (Figure 5). In gray is construction without limiters. The gray and colored bars show which system has a greater energy harvesting effectiveness (difference in the RMS values of the voltages induced on the piezoelectric electrodes Δ*u_RMS_*) for a given dimensionless excitation frequency.

To assess the effectiveness of energy generation, an indicator based on the difference in the RMS values of the voltages induced on the piezoelectric electrodes Δ*u_RMS_* for energy harvesting systems with and without motion limiters was used (Figure 9). At this point, we indicate that the adopted indicator is identified for a steady state of the system in a time window with a width of 50 periods of external excitation. We deal with the steady state of a dynamical system at the moment of extinction of transient processes and phenomena characterizing unstable periodic and chaotic solutions. The colors of the plotted RMS diagrams directly correlate with the corresponding characteristics of the potentials, which are depicted in the graphs (Figure 5). The values of the dimensionless frequency of the external load in which the design solution without bumpers shows a higher efficiency of energy harvesting in relation to the system with bumpers are marked in gray.

The results of numerical experiments that are carried out for the energy harvesting system with a two-well potential (Figure 9a) indicate that the use of mechanical bumpers improves the energy generation effectiveness in a relatively narrow band of variation of the control parameter ω ∈ [3, 4.5] (*p* = 0.1). With regard to the other values of ω, there are also high color peaks of the RMS difference. Nevertheless, in the case of the indicated band, we can talk about a homogeneous zone. With regard to the remaining values of the dimensionless excitation frequency, e.g., ω ∈ [7, 8.5] (*p* = 0.1), we deal with an alternating distribution of gray and colored peaks. In other words, for one frequency, the system with bumpers shows a better ability to generate energy than the design solution without bumpers, while a relatively small frequency disturbance reverses the situation. In such situations, the use of motion limiters acts as a fuse preventing excessive deformations of the flexible cantilever beam, which may lead to structural damage in the long term. Regardless of the width of the “zone of free motion” and the substitute value of the bumper stiffness, it basically does not change its position in the frequency spectrum. However, in the range of low values of the dimensionless excitation frequency ω < 1.3, regardless of the considered design solution, the use of motion limiters is pointless because the identified differences in the RMS values of the voltage induced on the piezoelectric electrodes take values close to zero. Such values indicate that we are dealing with small orbits of solutions that are located inside the potential well. Doubling the level of external load affecting the two-well system with mechanical bumpers drastically reduces the effectiveness of energy harvesting. Only in very narrow bands located in the range of ω ∈ [1.5, 2] does the use of limiters have a beneficial effect.

If the design solution of the energy harvesting system is based on the three-well potential (Figure 9b), the use of bumpers is possible in relatively narrow bands. However, at a low level of external excitation *p* = 0.1, in the bands ω ∈ [0, 3] and ω ∈ [6.5, 9], no improvement in the effectiveness of energy generation through the use of mechanical limiters is observed. Correcting their mechanical characteristics and location also does not improve the situation. Large differences in RMS values of the voltage induced on the piezoelectric electrodes occur in the range of high excitation frequencies ω > 9. Nevertheless, they do not indicate whether we deal with periodic solutions, which are characterized by significantly higher energy effectiveness in relation to unpredictable solutions. Considering the variable heights of individual Δ*u_RMS_* peaks, we can suspect the existence of chaotic or quasiperiodic solutions. The answer we deal with can be obtained at the point of additional numerical simulations, the results of which are included in the further part of the work. As is the case with the design solution based on the two-well potential, doubling the dimensionless amplitude of mechanical vibrations to the level of *p* = 0.2 means that the bumpers used in the system fulfill the function of protecting against excessive vibrations of the flexible cantilever beam.

The greatest energy benefits from the use of mechanical bumpers are recorded in the case of the energy harvesting system based on the four-well potential (Figure 9c). This situation takes place because a relatively small external load (*p* = 0.1) can cause the “knockout” of the solution trajectory from relatively shallow potential wells. In the range of variability ω ∈ [2.25, 7.25], we are dealing with a homogeneous band, excluding from it the area of variability ω ∈ [2.75, 4.25] of the dominance of the construction solution with motion limiters over the solution without them. The uniform distribution of differences in RMS values of the voltage induced on the piezoelectric electrodes suggests the existence of periodic solutions. Nevertheless, limiting the spacing of the bumpers with a simultaneous reduction of the equivalent stiffness (diagrams drawn in red and green) causes unpredictable solutions. As is the case in the two previously analyzed design solutions, in the range of low excitation frequencies ω < 1.5, no impact of the applied bumpers on the improvement of energy harvesting effectiveness is observed. Increasing the external load to the level *p* = 0.2 makes effective energy harvesting possible in a narrow band ω ∈ [1.3, 1.8] when dealing with rigid bumpers (diagram drawn in blue). A direct comparison of RMS difference diagrams suggests that reducing the stiffness of the bumpers while limiting the “zone of free motion” increases the effectiveness of energy harvesting (the area highlighted in gray decreases).

**Figure 10:**
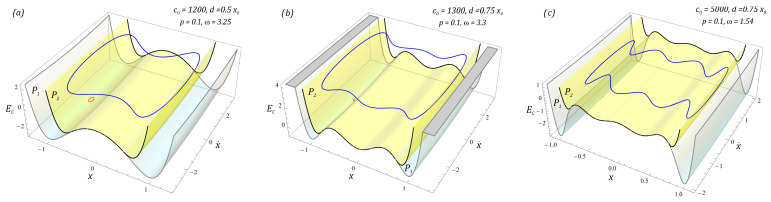
The results of computer simulations showing periodic solutions with and without motion limiters in systems with the following potential: (**a**) two-well, (**b**) three-well, (**c**) four-well. Potentials characterizing the system without bumpers (*P*_1_ surfaces) and with bumpers (*P*_2_ surfaces) are shown. Orbits without limiters in red; with, in blue.

Exemplary images of stable periodic solutions limited to a single case corresponding to each potential are shown in Figure 10. This is because, in the case of stable periodic solutions which are provided in the form of large orbits circling all the potential wells, the dimensionless excitation frequency affects the energy trajectory in steady state. However, for high-energy orbits, its shape resembles a rectangle with rounded tops. The selected results presented in the graphs (Figure 10) clearly illustrate the beneficial effect of the use of mechanical limiters on energy harvesting.

The results of computer simulations are presented in the form of spatial orbits depicted against the background of potentials characterizing the system without (*P*_1_ surfaces) and with bumpers (*P*_2_ surfaces). In the case of structural solutions without bumpers, the orbits of stable periodic solutions plotted in red are located inside the external potential wells and are characterized by small vibration amplitudes of flexible cantilever beams. On the other hand, steady-state phase streams of energy harvesting systems with mechanical limiters are plotted in blue. It is worth noting that the appropriately selected characteristics of the bumpers work to minimize the effect of attracting the phase stream through deep wells from which they cannot escape at a low level of external load affecting the system. Orbits can leave the potential wells when dealing with a chaotic solution or a sufficiently high level of mechanical vibrations affecting the system.

**Figure 11:**
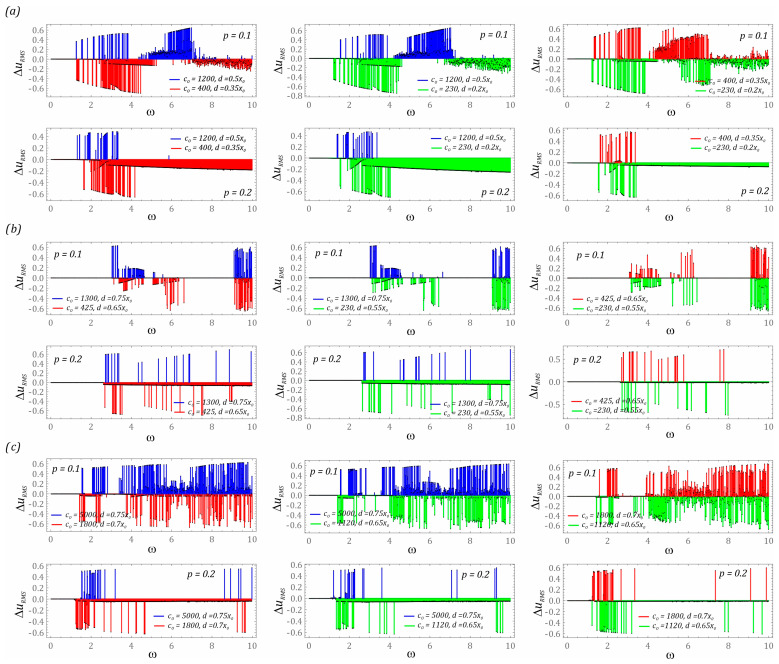
Comparison of the energy harvesting effectiveness of different mechanical characteristics of motion limiters in the following systems: (**a**) two-well, (**b**) three-well, (**c**) four-well. The colors of the plotted diagrams directly correlate with the corresponding characteristics of the potentials, which are depicted in the graphs (Figure 5). The colored bars show which limiter stiffness characteristics have greater energy harvesting effectiveness (difference in the RMS values of the voltages induced on the piezoelectric electrodes Δ*u_RMS_*) for a given dimensionless excitation frequency.

In the last stage of the model research, a comparison of the energy harvesting effectiveness of different bumper configurations in the analyzed design solutions is presented. Numerical experiments are carried out with reference to the same model assumptions as those adopted during computer simulations, the results of which are presented in the graphs (Figure 9). The results of the numerical simulations are presented in the form of diagrams of differences in the RMS values of the voltage induced on the piezoelectric electrodes (Figure 11). It is worth noting that, regardless of the potential characterizing the energy harvesting system, in the range of low values of the dimensionless excitation frequency ω < 1.5, the configuration and characteristics of the bumper do not have a significant impact on the harvester effectiveness (the difference Δ*u_RMS_* assumes values close to zero). This situation is also observed in the range of higher load levels affecting energy harvesting systems. In the case of the system based on the three-well potential, with the lack of sensitivity due to the configuration and characteristics of the bumper, we deal within the variability bands ω ∈ [0, 3] and ω ∈ [7, 9].

For the system based on the two-well potential (Figure 11a) in the range of low values of dimensionless mechanical vibration amplitudes (*p* = 0.1), it is preferable to use bumpers with rigid characteristics and a relatively wide “zone of free motion”. The frequency of the external load affecting the energy harvesting system should be in the range ω ∈ [5, 7].

On the other hand, in the range of higher values ω > 7, much better results will be obtained by using bumpers with soft mechanical characteristics and relatively narrow “zones of free motion”. Soft and narrow “zone of free motion” allows for more effective energy harvesting when the system is subjected to higher dynamic loads (*p* = 0.2). In the variability band ω ∈ [1, 4.5] (*p* = 0.1), the colors assigned to the individual characteristics of the bumpers are arranged alternately. Such a distribution of the diagrams of differences in RMS values of the voltage induced on the piezoelectric electrodes show a strong sensitivity to the configuration and characteristics of the bumpers used. This behavior of the system is also observed in the case of a design solution based on the three-well potential (Figure 11b). Taking into account the results presented in the diagrams (Figure 9b), it can be concluded that the use of bumpers in the considered three-well system has a positive effect on improving the effectiveness of energy harvesting in very narrow bands of variability of the dimensionless frequency of the external excitation (*p* = 0.1).

The configuration and mechanical characteristics of the motion limiters (bumpers) are highly sensitive due to the change in the value of the control parameter in the energy harvesting system with a four-well potential (Figure 11c). Such dynamic properties are determined by the characteristics of the potential, which is characterized by very shallow internal wells. In addition, such behavior of the system is significantly influenced by the number of coexisting solutions, which—in the case of multi-well systems—is definitely higher than in systems with a smaller number of wells. In this system, there is basically no relatively wide homogeneous zone in which it would be possible to indicate the benefits resulting from the use of a dedicated configuration and characteristics of the bumpers. The situation changes significantly if the level of load affecting the energy harvesting system increases (*p* = 0.2). At higher levels of external mechanical vibration in the identified diagrams, it is possible to distinguish homogeneous bands where effective energy generation takes place. As in the other analyzed design solutions, and also in the case of a system with a four-well potential, it is advantageous to use bumpers whose mechanical properties are mapped with soft characteristics.

The results of computer simulations published so far illustrate the dynamic properties of the tested design solutions of energy harvesting systems in a qualitative manner. On their basis, it is possible to assess the nature of the system responses that are induced in a wide range of variability of the control parameter ω. Considering the quantitative assessment of the energy harvesting effectiveness, the results of the model tests were presented in the form of bar charts showing the power and effective voltage recorded on the piezoelectric electrodes. For this purpose, indicators were used, which were defined as the area under the diagrams of power and effective voltage values:

(8) $$\begin{array}{c} \begin{array}{cc} A_{P}=\int_{\omega_{i}}^{\omega_{j}}\kappa \cdot u^{2}d\omega , & A_{U}=\int_{\omega_{i}}^{\omega_{j}}ud\omega . \end{array} \end{array}$$

**Figure 12:**
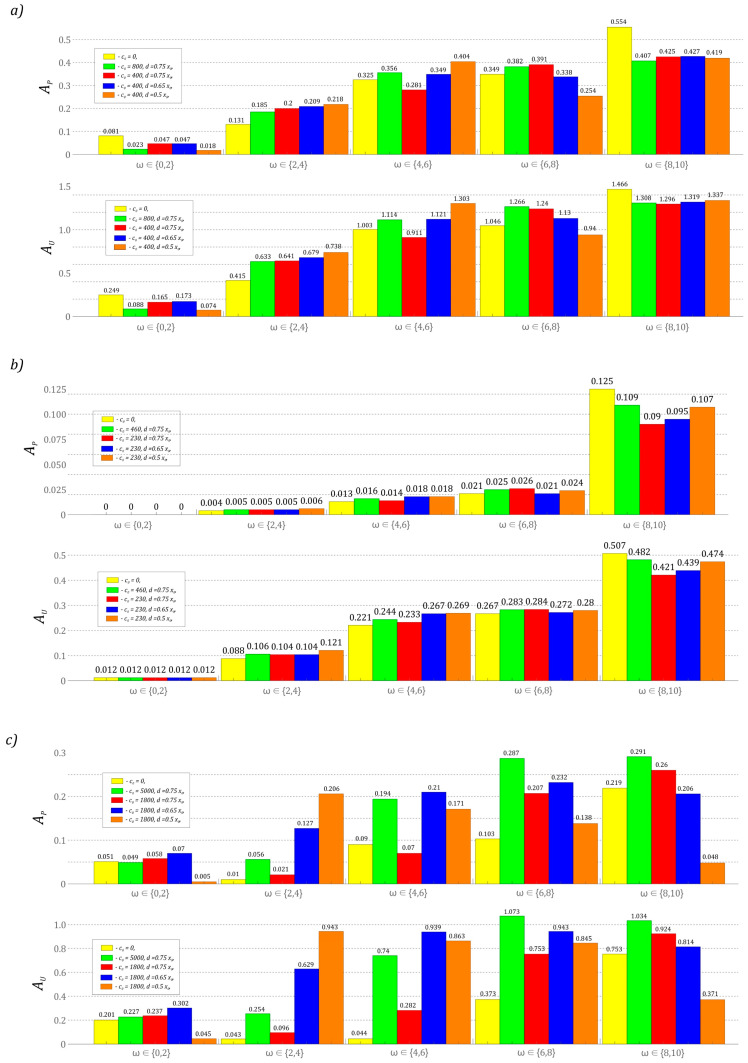
Quantitative results of computer simulations showing the effectiveness of energy harvesting in the system: (**a**) two-well, (**b**) three-well, (**c**) four-well. The bars highlighted in yellow correspond to the design solutions which do not include motion limiters. The colors of the individual bars represent the system characteristics described in the legend. Simulations were performed for zero initial conditions.

Through them, it is possible to quantify the impact of the mechanical characteristics of the stoppers on the energy harvesting effectiveness. At this point, it is worth noting that regardless of what indicator is taken as the baseline (power or RMS voltage), forecasting the effectiveness of the tested solutions provides similar results (Figure 12). The results of numerical simulations presented in the graphs were carried out for low levels of mechanical vibrations *p* = 0.1. For larger values of the dimensionless vibration amplitude *p*, the defined coefficients assume correspondingly higher values. The results presented in the graphs were obtained assuming zero initial conditions and fixed positions of mechanical stoppers in relation to the potential characteristics reflecting the dynamic properties of the tested solution. The bars highlighted in yellow correspond to the design solutions of the energy harvesting system, which does not include motion limiters.

The presented results clearly show that the introduction of mechanical bumpers in the design solution significantly improves the energy harvesting effectiveness in the system with the potential represented by four wells (Figure 12c). This property basically applies to the entire analyzed range of variability of the control parameter. Only in the case of very low ω ∈ [0, 2] and very high ω ∈ [8, 10] external load frequencies does the use of motion limiters drastically reduce the energy generation (bars highlighted in orange). Such a large increase in the energy harvesting effectiveness in the four-well system is directly determined by the height of the internal potential barriers, which are definitely lower in relation to the two-well (Figure 12a) and three-well (Figure 12b) systems. In relation to these systems, the use of stoppers does not look so impressive. For the case of high excitation frequencies ω ∈ [8, 10], one can even speak of a limitation of the energy generation. On the other hand, in the intermediate bands of the variability of the control parameter ω, we do not deal with such a large improvement as in the four-well system.

It is worth noting that, for the system represented by the three-well potential, effective energy harvesting takes place in the range of high excitation frequency values ω ∈ [8, 10] (Figure 12b). This situation is caused by excitation for zero initial conditions of periodic solutions represented by a large orbit circulating around the potential well. This case shows, in principle, a negligible ability to harvest energy in the range of low frequencies of the external load ω ∈ [0, 2]. A direct comparison of the values of the identified indicators (8) shows that the three-well system is characterized by the lowest effectiveness of energy harvesting, despite the fact that the height of the potential barrier is much lower than the two-well system. This situation is directly related to the width of the potential. At very low levels of mechanical vibrations affecting the three-well system, the potential width is so large that the external excitation does not provide enough energy to achieve a periodic orbit around all wells. The results of computer simulations indicate that the distance measured between the contact surfaces of the bumpers should be taken as equal to *d* = 0.75*x*_0_, because their close proximity reduces the effectiveness of energy harvesting (graph bars marked in red, blue and orange). Only in the case of the system represented by the three-well potential is the influence of the distance measured between the bumpers basically not significant.

## 4. Summary and Conclusions

The use of the Fibonacci hyperbolic sine is a simple and effective numerical tool for mapping the non-linear properties of bumpers in energy harvesting systems. The use of the Fibonacci function makes it possible to simplify the mathematical model because the mechanical characteristics of the bumpers can be modified by changing only one parameter. This is a significant simplification compared to the model based on a polynomial function. In addition, the model represented by straight lines is an idealized description of the functioning of the limiter. It does not fully reflect the nature of cooperation with the free end of the beam. The use of the Fibonacci function makes it possible to take into account the elastic deformations of the bumper at the moment of contact with the beam. In the case of a bumper model based on a linear function with a dead zone, we are not able to take this into account.

The most effective impact of the use of bumpers can be observed when dealing with deep external wells. The width of the “zone of free motion” should be limited by the minima of the outer wells and the maxima of the outer saddles. By means of mechanical limiters, it is possible to minimize the influence of the depth of external wells. In addition, the use of mechanical bumpers in the design solutions of energy harvesting systems reduces the vibration amplitude of flexible cantilever beams.

The use of mechanical bumpers in the design solution allows us to increase the energy harvesting effectiveness when the system is subjected to relatively small dynamic loads and the use of motion limiters does not affect the presence of the transient chaos phenomenon. The distance measured between the contact surfaces of the bumpers should be taken as equal to *d* = 0.75*x*_0_, because their close proximity reduces the effectiveness of energy harvesting.

The presented simulation results concern tests aimed at providing information about the possibility of designing a laboratory stand and the possible results. Now, we are at the stage of preparing experiments. Model studies provide information on how to plan the course of an laboratory experiment. In addition, our simulation results consider the system in a wide range of parameters, while laboratory experiments will be limited to selected configurations characterized by the best energy harvesting effectiveness.

## Figures and Tables

**Table 1:** Geometric and physical parameters characterizing the tested systems.

Name	Symbol	Value		
Inertial element (mass) loading the beam	*m*	0.01 kg		
Energy losses in the cantilever system	*b_B_*	0.02 Nsm^−1^		
Cantilever beam stiffness	*c_B_*	15 Nm^−1^		
Scaling parameters	*x*_0_	0.0253 m	0.03 m	0.0307 m
Physical parameters defining potential barriers	*c*_1_ *c*_2_ *c*_3_ *c*_4_	144 Nm^−1^ 35 × 10^7^ Nm^−5^	243 Nm^−1^ 1.35 × 10^6^ Nm^−3^ 1.2 × 10^9^ Nm^−5^	210 Nm^−1^ 2.7 × 10^6^ Nm^−3^ 7.8 × 10^9^ Nm^−5^ 5496 × 10^9^ Nm^−7^
Load resistance	*R_p_*	1.1 MΩ		
Piezoelectric capacity	*C_P_*	72 nF		
Electromechanical constant of piezoelectric converter	*k_P_*	3.985 × 10^−5^ NV^−1^		

## Data Availability

Data are contained within the article.
